# Case of Aneurism of the Aorta

**Published:** 1816-08

**Authors:** 

**Affiliations:** Surgeon


### Case of Aneurism of the Aorta.

By Mr. Howship, Surgeon.

Richard Smith, mason, a strong muscular man, about fifty years of age, first complained of a pain in the small of his back, in January, 1814, almost immediately after lifting a heavy burthen. This pain was of a spasmodic kind, returned by fits, and occasionally with great violence, which opiates alone were capable of relieving.

During the first six months, these attacks of pain were confined to the back; after this period, however, he began to feel his complaint move towards the left side, where some degree of external swelling soon made its appearance, just below the false ribs. As this swelling extended itself, he found the pains were no longer confined either to the back or the side, but were felt throughout nearly the whole extent of the abdomen.

When he was first attacked, he was generally able to work at his business, but always found the symptoms increase after lifting any heavy weight. He was also frequently affected with a shortness of breath, a troublesome cough, and a difficulty in making water, which symptoms, added to the pains he felt, induced him to believe he had the gravel. The day before he died, his urine was tinged with blood.

On the 11th of June, 1815, he was extremely ill, confined to his bed, and had all the appearance of a dying man. On laying the hand upon the swelling, a very evident and strong pulsation was felt. In the course of the second night afterward he died. On the following day, permission was obtained to open the body.

EXAMINATION.

The external swelling upon the side had rather subsided, although the abdomen was hard and tense. Upon laying open the cavity of the belly, a large oblong prominent tumour was perceived on the left side, extending itself from the diaphragm to the brim of the pelvis. On a more near examination, the swelling was found to be situated within the circle of the colon, upon the left side of the spine, pushing the small intestines to the opposite side, and projecting so far forward as to be in contact with the peritoneum lining the anterior part of the abdomen.

The anterior and most prominent part of the tumour proved to be the left kidney, in a sound state, but removed from its natural situation by the remaining portion of the swelling, which was very large. Behind the kidney was found a considerable quantity of coagulated blood, that had diffused itself through the cellular membrane. This mass of coagulated blood was of considerable extent; and on being divided through, for the depth of an inch, a large bag was exposed, which proved to be an aneurism of the aorta. To obtain a better view of the disease, the crura of the diaphragm and root of the mesentery, together with the fat and glands that cover the aorta, were dissected away. This operation exposed the whole extent of the disease, which formed a large oblong tumour, extending over the lower vertebræ of the back and the upper vertebræ of the loins. This tumour was unequally divided by the aorta; that part which lay on the left side of the spine was by much the largest, measuring seven inches in length and five in breadth.

On laying open this tumour, its contents were principally fluid, or but recently coagulated blood; there were, however, two firm lamellated coagula enclosed within the parietes of the sac. It appeared, that the larger of these coagula had performed the office of a plug, in checking the effusion of blood from the sac through a large aperture that was discovered leading to the cellular membrane, in which the mass of coagulated blood had been previously found. That part of the tumour which lay on the right side of the spine was situated between the aorta and the right kidney, and was not larger than a small sized apple. The aneurismal swelling had, in a manner, embraced the anterior part of the spine, by being firmly connected to the sides of the vertebræ; and when this connection was separated, a large quantity of fluid, as well as coagulated blood, escaped. The coagulated blood had only the consistence of jelly, as if formed in articulo mortis.

To expose the inside of the sac more fully, and to obtain a view of the spine, the whole tumour was turned aside, when the cavity of the sac was found to be a smooth surface; and it appeared that the dilatation of the aorta had taken place backward, and to each side. The anterior part of the aorta was free from disease, except that a complete scale of ossification was found as large as a sixpence, formed in the cellular membrane between the coats of the artery, near the origin of the superior mesenteric artery. The enlargement had commenced about one inch above the origin of the cœliac artery, and terminated below, nearly opposite the point where the superior mesenteric artery begins.

Mill Street, Hanover Square, June 18, 1816.

